# One Step Closer: A Replicable Peer-Support Model to Enhance Wellbeing in Early-Career Medical Professionals

**DOI:** 10.1192/bjo.2026.11330

**Published:** 2026-06-30

**Authors:** Favour Adanna Chimere, Victor Nwamarae

**Affiliations:** NHS, Carlisle, United Kingdom

## Abstract

**Aims:**

To foster wellbeing, resilience, and professional integration through structured peer support, storytelling, and reflective practice, and to provide a model that can be replicated across clinical teams or patient support groups for mental health conditions.

Transitions in professional life–such as starting the UK Foundation Programme–are high-stress periods that can impact mental health, emotional resilience, and professional confidence, particularly for international medical graduates (IMGs). One Step Closer is an online community I founded to provide peer-led guidance, emotional support, and reflective learning for early-career doctors. Beyond supporting foundation doctors, the principles underpinning this model are applicable to healthcare teams, patient peer-support groups, and wider mental health interventions.

**Methods:**

The initiative combines online community engagement with reflective dialogue and storytelling shared via social media. Content focuses on professional preparedness, emotional resilience, and practical guidance during transitions into clinical practice. Participation from other foundation doctors enhances collective learning, normalizes emotional experiences, and strengthens community cohesion. The structured, peer-led approach is easily adaptable for workplace teams, multidisciplinary groups, or patient-led support networks in mental health care.

**Results:**

Participants reported enhanced confidence, sense of belonging, and access to emotional and practical support. The community facilitated reflective practice, resilience-building, and peer mentorship. By providing a structured yet flexible framework, the initiative demonstrates how small, intentional acts of support can improve wellbeing and integration, while being replicable across different professional or clinical contexts.

**Conclusion:**

One Step Closer exemplifies how peer-led storytelling and structured support networks can promote mental health, professional development, and reflective practice. The approach is scalable and adaptable to healthcare teams, patient support groups, or mental health interventions, offering a practical framework for enhancing wellbeing, representation, and peer support in diverse clinical and educational settings.

